# Use of machine learning to predict cognitive performance based on brain metabolism in Neurofibromatosis type 1

**DOI:** 10.1371/journal.pone.0203520

**Published:** 2018-09-07

**Authors:** Manuel Schütze, Danielle de Souza Costa, Jonas Jardim de Paula, Leandro Fernandes Malloy-Diniz, Carlos Malamut, Marcelo Mamede, Débora Marques de Miranda, Michael Brammer, Marco Aurélio Romano-Silva

**Affiliations:** 1 Instituto Nacional de Ciência e Tecnologia de Medicina Molecular, Universidade Federal de Minas Gerais, Belo Horizonte, Brazil; 2 Centro de Desenvolvimento da Tecnologia Nuclear, Comissão Nacional de Energia Nuclear, Belo Horizonte, Brazil; 3 Departamento de Anatomia e Imagem, Faculdade de Medicina, Universidade Federal de Minas Gerais, Belo Horizonte, Brazil; 4 Departamento de Pediatria, Faculdade de Medicina, Universidade Federal de Minas Gerais, Belo Horizonte, Brazil; 5 Department of Neuroimaging, Institute of Psychiatry, London, United Kingdom; 6 Departamento de Saúde Mental, Faculdade de Medicina, Universidade Federal de Minas Gerais, Belo Horizonte, Brazil; Banner Alzheimer’s Institute, UNITED STATES

## Abstract

Neurofibromatosis Type 1 (NF1) can cause a wide range of cognitive deficits, but its underlying nature is still unknown. We investigated the correlation between cognitive performance and specific patterns of resting-state brain metabolism in a NF1 sample. Sixteen individuals diagnosed with NF1 underwent 18F-FDG PET/CT brain imaging followed by a neuropsychological assessment. Principal component analysis was performed on 17 measures of cognitive function and a machine learning approach based on Gaussian Process Regression was used to individually predict the components that represented most of the variance in the neuropsychological data. The accuracy of the method was estimated using leave-one-out cross-validation and its significance through permutation testing. We found that only the first component could be accurately predicted from resting state metabolism (*r* = 0.926, p<0.001). Multiple and heterogeneous measures contribute to the first component, mainly WISC/WAIS Procedure and Verbal IQ, verbal memory and fluency. Considering the accurate prediction of measures of neuropsychological performance based on brain metabolism in NF1 patients, this suggests an underlying metabolic pattern that relates to cognitive performance in this group.

## Introduction

Neurofibromatosis type 1 (NF1) is a disorder caused by mutations in the neurofibromin gene, which affects the synthesis of a protein widely expressed and involved in many vital pathways. Its presentation is characterized by multiple *café-au-lait* spots, skin-fold freckling, cognitive and behavioral deficits, and benign or malignant tumors [1]. The cognitive and behavioral deficits represent the most widely reported cause of overall medical appointments in young NF1 patients, commonly resulting in academic failure and lower quality of life [2,3]. We have also recently reported that the impairments persist in elderly NF1 individuals [4]. Almost 80% of NF1 individuals show at least one of the following cognitive deficits: visuoperceptual deficits, poor motor coordination, inferior executive function performance, attentional disorder, lower IQ scores, and language delay [2,5].

The fact that a monogenic disorder can cause a wide range of cognitive deficits makes NF1 an interesting model to investigate structural, functional, and molecular pathways associated with complex behavior endophenotypes, using tools such as functional and molecular neuroimaging techniques. MRI studies show that brain abnormalities in NF1 include increased white matter volume, increased subcortical grey matter volume (e.g., thalamus, right caudate), decreased cortical grey matter density, T2 hyperintensities (T2H), macrocephaly, and reduced integrity of white matter microstructure [6–10]. Thalamic T2H and volume abnormalities in the corpus callosum, putamen, and amygdala were associated with cognitive deficits in NF1 [2,6,9]. However, limited data are available concerning 18F-Fluordeoxyglucose Positron Emission Tomography (18F-FDG PET) brain imaging in NF1. Balestri and colleagues used 18F-FDG PET to evaluate 4 NF1 patients and 9 controls with ages ranging from 10 to 20 years and found widespread cerebral hypometabolism [11]. Kaplan and colleagues found decreased thalamic metabolism and varying degrees of cortical inhomogeneity in their 18F-FDG PET study with 10 NF1 patients and 9 controls aged between 4 and 15 years [12]. Thalamic hypometabolism was also observed by Buchert and colleagues, who reported an average decrease around 8% in FDG retention in the thalamic region of NF1 patients around the fourth decade of life using a voxel-based analysis [13]. Apostolova and colleagues recently confirmed these findings in a matched case-control study with 50 NF1 subjects, reporting a 7.6% lower FDG uptake in the thalamic region of the NF1 group [14].

Considering the prevalence of cognitive deficits in NF1 and reports of 18F-FDG PET studies in this group, we aimed to investigate whether cognitive performance in subjects with NF1 is associated with specific patterns of brain metabolism. To answer this question, a machine learning approach based on Gaussian Processes was chosen.

## Subjects and methods

### Participants

#### NF1 group

In this study, a group of 16 individuals diagnosed with NF1 was investigated by a multidisciplinary assessment team at the Neurofibromatosis Outpatient Reference Center of Minas Gerais. The study was conducted according to the principles expressed in the Declaration of Helsinki. All subjects have given written informed consent, and the study was approved by the Human Ethics Committee of the Federal University of Minas Gerais (UFMG), in Belo Horizonte, Brazil and is registered under the number CAAE-01344212.2.0000.5149. For minor patients, written consent was obtained from their parents or guardians. The diagnosis was performed using guidelines proposed by the NIH [1]. Participants’ age ranged from 8 to 44 years and length of formal education from 2 to 16 years. Socioeconomic status ranged from low to middle (median monthly household income between U$348 and U$1,190). The 16 patients were selected from a group of 41 patients who underwent whole-body 18F-FDG PET/CT imaging due to symptomatic plexiform neurofibromas (to establish the risk of malignant progression of the disease) [15]. Six patients were not included in this study for not having brain image data, three patients did not perform the cognitive tests, one patient was too young (4 years old) for the available neuropsychological tests, three patients were excluded due to artifacts in their brain images (most likely due to movement between the CT and the PET affecting the attenuation correction), and 12 had incomplete neuropsychological data. None of the patients had a history of brain tumor or presented current evidence of a brain tumor after visual assessment of the PET and CT images by an experienced nuclear medicine physician. There was no specific control for medication use. From those patients taking medications, only one (female, 36 years) reported use of a psychotropic drug (antidepressant).

#### Control group

A group of 16 non-psychiatric, non-neurologic, and non-oncologic individuals matched by age, education, and gender was voluntarily submitted to the neuropsychological evaluation and compared to the NF1 group. Exclusion criteria included history or current evidence of psychosis, autism, brain disorders, or any genetic or medical disorder associated with cognitive impairment. None of the controls reported previous or current use of psychotropic drugs. All controls gave written informed consent to participate in this study.

### Neuropsychological assessment

Participants underwent a comprehensive neuropsychological assessment to measure intelligence, attention/processing speed, visuospatial abilities, episodic memory, fine motor coordination, and executive functions. All instruments were adapted and validated for the Brazilian context. They can be used by children older than seven years and adults, as well as by subjects from different educational backgrounds.

*Intelligence*—General intellectual functioning was assessed by the third version of the Brazilian Wechsler Intelligence Scales (WAIS-III for adults and WISC-III for children) verbal (VIQ), procedure (PIQ) and full-scale (IQ) intelligence quotients [16,17].

*Fine Motor Coordination*—The Nine-Hole Peg Test was performed as a measure of finger dexterity [18]. It measures the time (in seconds) needed to complete the test with the dominant and the non-dominant hands.

*Visuospatial Abilities*—The Rey-Osterrieth Complex Figure Test was implemented as a measure of the visuospatial cognitive domain [19].

*Episodic Memory*—We selected both verbal and non-verbal episodic memory tests since NF1 patients might show a discrepancy between these two functional aspects [5]. Immediate (A6) and delayed recalls (A7) of the Brazilian-Portuguese version of the Rey Auditory-Verbal Learning Test (RAVLT) were taken as measures of verbal episodic memory [20]. Immediate (~3 minutes) and delayed (30 minutes) recalls of the Rey-Osterrieth Complex Figure (ROCF) were adopted as non-verbal memory measures.

*Language*—Assessment of this cognitive domain was performed by a Category Fluency Test (“animals”) [21], and by Lexical Fluency Test letters (F, A, and S) [22].

*Attention/Processing Speed*—The Five Digits Test (FDT) [23] is a numeric-Stroop paradigm designed to assess attentional processes. We adopted the first component of the test (FDT-Decoding), which involves reading/naming of numbers ranging from 1 to 5, as a measure of Automatic Attentional Processing and Processing speed [24]. The Inhibitory Control was investigated by the Inhibiting component of the Five Digits Test (FDT—Inhibiting), which involves inhibition of an automatic attentional routine in favor of a controlled one, demanding executive control.

*Executive Functions*—For working memory evaluation, we used the Digit Span according to the WAIS-III or WISC-III, and the Corsi block-tapping task [25]. The product of the span length and the number of correctly remembered trials, both for the Corsi test and the Digit Span, were taken into account [26]. The Tower of London Test [27] was used as a measure of Planning Skills.

The neuropsychological examinations were conducted between February 2012 and October 2013 and took place no longer than one month after the brain image acquisition.

### Demographic and neuropsychological data analysis

We analyzed differences in the neuropsychological performance between the NF1 group and the control group. Descriptive statistics were generated for gender, age, education, socioeconomic level, and neuropsychological data. The Student *t*-test was carried out to investigate group differences in continuous variables, and the Chi-square test was used to assess differences in frequency of dichotomous variables (i.e., gender). Effect sizes of observed differences were also calculated (Cohen’s *d*). Measures from the Nine-Hole Peg Test (dominant and non-dominant hands) were not originally normally distributed, thus scores were normalized through a log transformation.

### Image acquisition

Resting-state 18F-FDG PET/CT brain images were acquired in a GE Discovery 690 (GE Healthcare, Millwalke, EUA) scanner as part of a whole-body scan. Patients had at least six hours of fasting before the exam. After an intravenous bolus injection of 5.18 MBq/kg of 18F-FDG, patients rested for 50 minutes in a quiet and dark room, with minimum stimuli. PET brain images were acquired subsequently, as a separate volume from the rest of the body, with a total acquisition time of 10 minutes, and reconstructed in a 192x192x47 matrix using the OSEM (Ordered Subsets Expectation Maximization) algorithm, with 2 iterations and 20 subsets. Attenuation correction was performed using the CT image.

### Image processing

PET images were spatially processed using the Statistical Parametric Mapping toolbox (SPM8, Wellcome Trust Centre for Neuroimaging, 2008) implemented within Matlab 7.12.0 (MathWorks, Natick, MA, USA). Initially, a gross manual image reorientation and approximate definition of the image center point was applied. All images were then spatially normalized onto a 18F-FDG PET template in MNI space [28] and smoothed by a 12 mm FWHM Gaussian kernel, yielding a 91x109x91 volume per subject. To increase processing speed, this image was then downsampled to a 45x54x45 volume by averaging the values of 4 adjacent voxel. This was a necessary step for permutation testing during image analysis and in our tests didn’t significantly impact prediction accuracy. SPM’s brain mask was used to segment the brain from this volume, resulting in the selection of 33 570 voxels. Finally, these brain voxels were scaled by their global mean to account for differences in global signal between subjects [29]. The information extracted from each 18F-FDG PET scan is a single 33 570 × 1 data vector that “summarizes” each patient’s brain metabolism.

### Image data analysis

Our image data analysis involved investigating the correlation between neuropsychological variables and brain metabolism in NF1 patients. The traditional method to do this analysis would be by using the General Linear Model [30] to evaluate voxel-by-voxel whether the metabolism at a certain location could be well reconstructed by a combination of several regressors, including nuisance factors. In recent years though, machine learning (ML) has emerged as an alternative approach to standard neuroimaging analysis [31–33]. There are two important differences between ML and traditional mass-univariate statistics. First, the primary focus of ML is to make predictions, rather than explain how independent variables affect a certain outcome. Secondly, ML is a multivariate approach at the single subject level, i.e. predictions are made based on the distributed pattern of effects across the whole brain [34]. This is particularly useful when dealing with a high number of predictor variables (in our case, more than 33 thousand), especially where the variables are correlated, and there are many complex interactions between them.

Simply put, ML works by “learning” from training data to create a model that maps an input (e.g. set of PET images) to an output (e.g. clinical data). The primary outcome measure of ML is how well this model generalizes to new data. To assess this, the ML algorithm can be iteratively trained on a subset of the data and then tested on the remaining “unseen” data (cross-validation). This strategy also avoids overfitting, which occurs when a model starts “memorizing” training data rather than “learning” to generalize from a trend.

Different machine learning algorithms would be suited to solve our regression problem [35]. We opted to use Gaussian Processes (GP) because it provides a principled, practical and probabilistic approach to learning in kernel machines, with the advantage of automatic tuning of the kernel parameters from the training data via type-II maximum likelihood [36]. Furthermore, GP models have been successfully applied to neuroimaging, in various clinical settings, including prediction of pain states, symptom severity, cognitive and disease states [37–41].

A GP is a generalization of a Gaussian (normal) probability distribution (which describes a finite-dimensional random variable) to functions. Thus, it extends multivariate Gaussian distributions to infinite dimensionality. By using GP as a prior distribution over the functional feature space, it is possible to use Bayesian inference to predict clinical outcomes or disease status [36].

For our analysis, we used the Gaussian Process regression (GPR) implementation available within *kernlab* R library [42,43]. “Vanilladot” was the linear kernel function selected, and the initial noise variance and tolerance of termination criteria were both set to 0.001.

Before performing the GPR analysis, two issues had to be addressed regarding the PET and neuropsychological data. Firstly, patients presented a considerable variation in age and since it is known that age influences global and regional brain metabolism [44,45], voxel values were processed to reduce the effect of age. This was done by applying a voxelwise linear regression and then performing GPR on the residuals. Secondly, since many of the 17 neuropsychological tests overlap in the cognitive processes they measure, we performed principal component analysis (PCA) in R to select the components that explained most of the variance in the data. For this, neuropsychological data was centered and scaled, and R’s *prcomp* function was used to extract the principal components. GPR was then performed on these components.

Leave-one-out cross-validation (LOOCV) was used to access the method’s accuracy. The algorithm was first trained on *n-1* subjects using the imaging data and the values of the selected principal component, and then predicted the value of the removed subject using only the image. This process was repeated *n* times so that prediction was performed for each subject. Predicted values were plotted against observed ones, and the Pearson correlation coefficient was calculated. To estimate the significance of the correlation, the LOOCV was repeated a thousand times with permutation of the input values [46]. Weight maps containing the most relevant voxels for prediction were produced for principal components with a significant correlation between predicted and observed values. These weight maps contain the average weight of each feature during the LOOCV.

## Results

Sixteen NF1 subjects from 8 to 44 years old were enrolled in this study. We started by establishing that the selected subjects were representative of the cognitive performance expected in NF1 patients by calculating the significance and magnitude of differences in neuropsychological tests compared to controls. We also evaluated differences in demographic characteristics to account for possible biases in the data. Table 1 shows the demographic and neuropsychological differences between NF1 patients and the control group. There were no differences between groups regarding age (p>0.05), education (p>0.05), SES (p>0.05), and gender (χ^2^(1, N = 32) = 1.166, p = 0.280). For neuropsychological variables, only large differences between groups reached statistical significance. The NF1 group had a significantly inferior performance compared to controls in intelligence (full scale IQ) and verbal short-term memory (i.e., Digit Span forward) tests. Group differences with medium effects were observed in measures of dexterity (9HPT dominant hand), visuoconstructive abilities (ROCF copy), short-term verbal and visual episodic memory (immediate recalls of RAVLT and ROCF) and visual working-memory (Corsi Block-Tapping Test forward and backward) with the NF1 group showing worse performance than controls. Small differences between NF1 patients and the control group were noticed in long-term episodic memory (delayed recalls of RAVLT and ROCF), verbal fluency, automatic (FDT-Decoding) and controlled (FDT-Inhibiting) attention processes, and planning (Tower of London). The statistical power of this analysis was of 82% for detecting large effects, 68% for detecting medium effects, and 53% for detecting small effects.

**Table 1:** Demographic and cognitive differences between groups (t-tests).

Domain	Measure	Control^a^ (n = 16)	NF1^a^ (n = 16)	*p*	*d*
Age (years)	**-**	25.56 (16.94)	23.75 (11.77)	0.728	0.13
Education (years)	**-**	9.06 (4.07)	8.69 (4.05)	0.796	0.10
Socioeconomic Status	CCEB	22.50 (5.85)	18.94 (7.02)	0.129	0.57
Intelligence (IQ)	WAIS-III/WISC-III (IQ)*	107.81 (5.64)	91.19 (15.28)	0.003	1.38
	WAIS-III/WISC-III (VIQ)*	108.44 (6.38)	92.44 (15.40)	0.003	1.34
	WAIS-III/WISC-III (PIQ)*	107.19 (6.88)	91.13 (15.27)	0.002	1.35
Finger Dexterity	9HPT (*dominant hand*)	0.79 (0.23)	0.89 (0.30)	0.327	-0.38
	9HPT (*non-dominant hand*)	0.87 (0.33)	0.91 (0.20)	0.685	-0.16
Visuospatial Abilities	ROCF (*Copy*)	30.38 (6.21)	26.81 (8.36)	0.181	0.50
Memory	RAVLT (A6 *Immediate Recall*)	10.06 (3.60)	8.31 (2.57)	0.125	0.58
	RAVLT (A7 *Delayed Recall*)	9.06 (3.00)	8.63 (2.50)	0.657	0.16
	ROCF (*Immediate Recall)*	17.94 (6.83)	12.94 (9.14)	0.090	0.64
	ROCF (*Delayed Recall*)	17.06 (6.34)	14.19 (6.77)	0.225	0.45
Language	VFT (Semantic)	15.63 (3.56)	15.00 (5.19)	0.694	0.15
	VFT (FAS)	29.75 (13.98)	27.25 (12.50)	0.598	0.19
Attention	FDT (Part 1-*Decoding*)	25.44 (8.73)	26.81 (7.09)	0.628	-0.18
	FDT (Part 3-*Inhibiting)*	53.19 (23.93)	52.19 (14.53)	0.999	0.00
Short-term/Working Memory	DST (*forward*)*	56.81 (27.26)	28.38 (12.50)	0.001	1.39
	DST (*backward*)	18.75 (11.54)	18.81 (10.09)	0.987	-0.01
	CBTT (*forward)*	45.75 (16.72)	38.13 (21.28)	0.269	0.41
	CBTT (*backward*)	36.81 (32.44)	20.38 (21.36)	0.101	0.62
Planning	TOL	29.56 (3.16)	28.69 (3.91)	0.492	0.25

* = p≤ 0.01.

Standard Deviations appear in parentheses.

^a^Means and Standard Deviations for non-transformed data.

CCEB = Brazilian Criterion of Economic Classification; WAIS-III/WISC-III = Wechsler Intelligence Scales (third editions); 9HPT = Nine Hole Peg Test (values log transformed); ROCF = Rey-Osterrieth Complex Figure Test; RAVLT = Rey Auditory Verbal Learning Test; VFT = Verbal fluency test; FDT = Five Digit Test; DST = Digit Span Test; CBTT = Corsi Block-Tapping Test; TOL = Tower of London.

**Fig 1:**
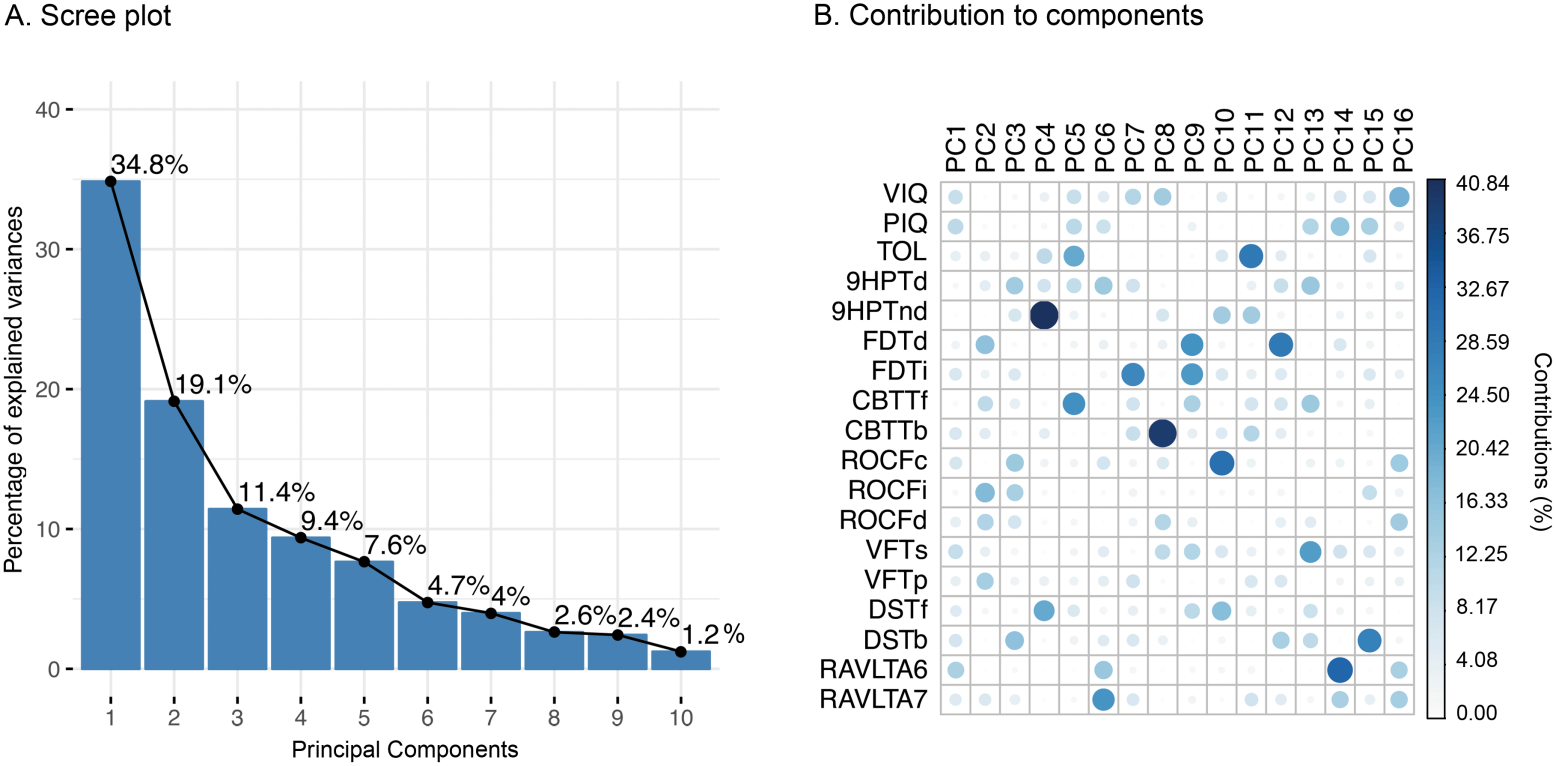
Principal component analysis on neuropsychological data. A. Scree plot containing the variance explained by each of the principal components. B. Contribution of variables to the principal components showing how much each variable contributes to the principal components. Note: VIQ = Verbal IQ; PIQ = Procedure IQ; TOL = Tower of London; 9HPT = Nine Hole Peg Test (values log transformed); FDT = Five Digit Test; CBTT = Corsi Block-Tapping Test; ROCF = Rey-Osterrieth Complex Figure Test; VFT = Verbal Fluency Test; DST = Digit Span Test; RAVLT = Rey Auditory Verbal Learning Test.

To establish whether the cognitive performance in NF1 patients could be correlated to a specific metabolic pattern, we used GPR to predict the principal components of the neuropsychological variables from brain metabolism images of the 16 NF1 patients. The explained variance for the ten most relevant principal components is depicted in Fig 1A. How much of each variable is represented on each component is depicted in Fig 1B.

**Fig 2:**
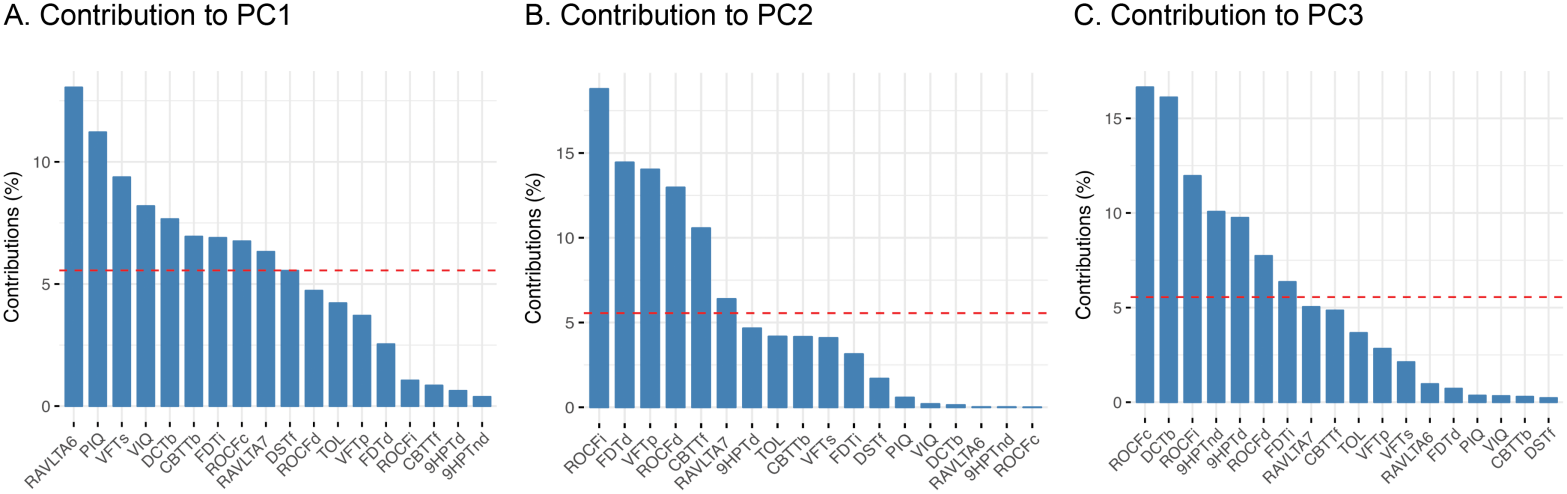
Contribution of neuropsychological measures to principal components. The red dashed line indicates the expected average contribution. Measures that contribute more than the expected average are important to that component. Note: VIQ = Verbal IQ; PIQ = Procedure IQ; TOL = Tower of London; 9HPT = Nine Hole Peg Test (values log transformed); FDT = Five Digit Test; CBTT = Corsi Block-Tapping Test; ROCF = Rey-Osterrieth Complex Figure Test; VFT = Verbal Fluency Test; DST = Digit Span Test; RAVLT = Rey Auditory Verbal Learning Test.

The first three components explain 65,3% of the variance in the neuropsychological data. How much each neuropsychological measure contributes to the first three components is detailed in Fig 2. The first component seems to represent general intelligence/verbal ability processes, including IQ measures, verbal memory and verbal fluency. Component two seems to represent more attentional/visuospatial processes, including visuospatial memory, simple processing speed and visuospatial working memory. Component three is represented by measures of motor coordination, visuospatial abilities and executive functions.

**Fig 3:**
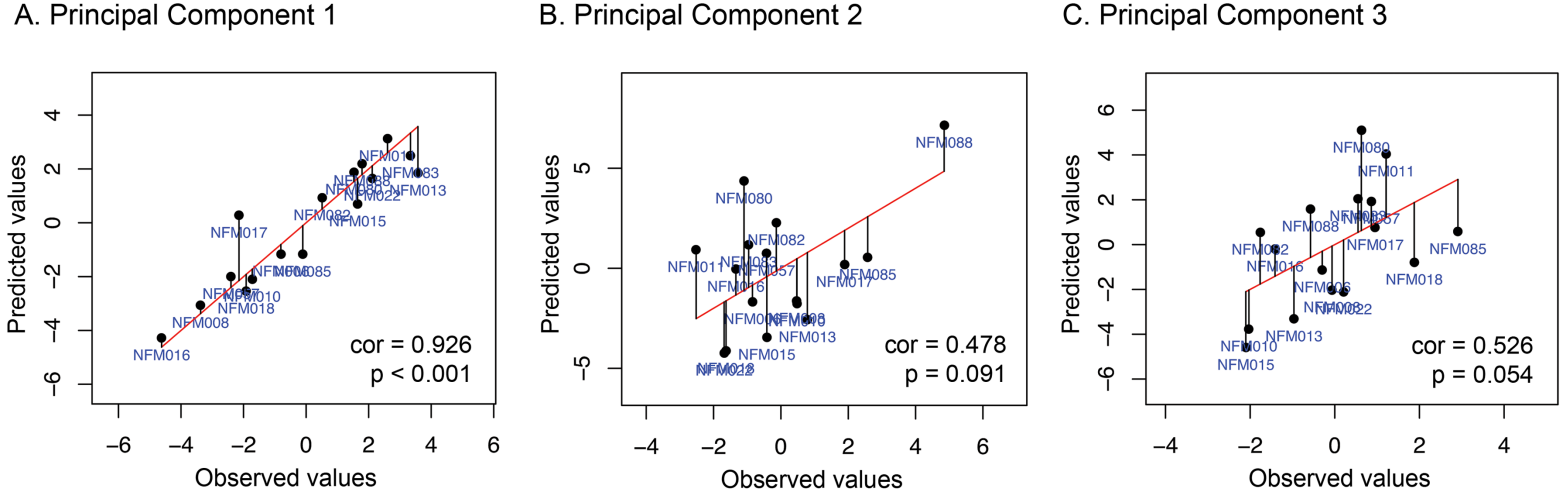
Predicted values plotted against observed values. Black points represent the value of the variable predicted during the LOOCV with the text in blue corresponding to the subjects’ ID; the red line represents the scenario where predicted values exactly match the observed ones, so that black vertical lines correspond to individual prediction errors. cor = Pearson’s product moment correlation coefficient.

Since the first three principal components explain a significant amount of the variance in the neuropsychological data, each one of these components was predicted individually through LOOCV and the Pearson correlation coefficient between predicted and observed values was calculated. Permutation testing was performed by randomly permuting values of the measured component (i.e. breaking the relationship between images and associated values). By repeating the permutation a thousand times, a p-value up to 0.001 can be estimated. Fig 3 shows the results for all tested variables. Only the first component (PC1) showed a significant correlation between observed and predicted values (*r* = 0.926, p<0.001).

**Fig 4:**
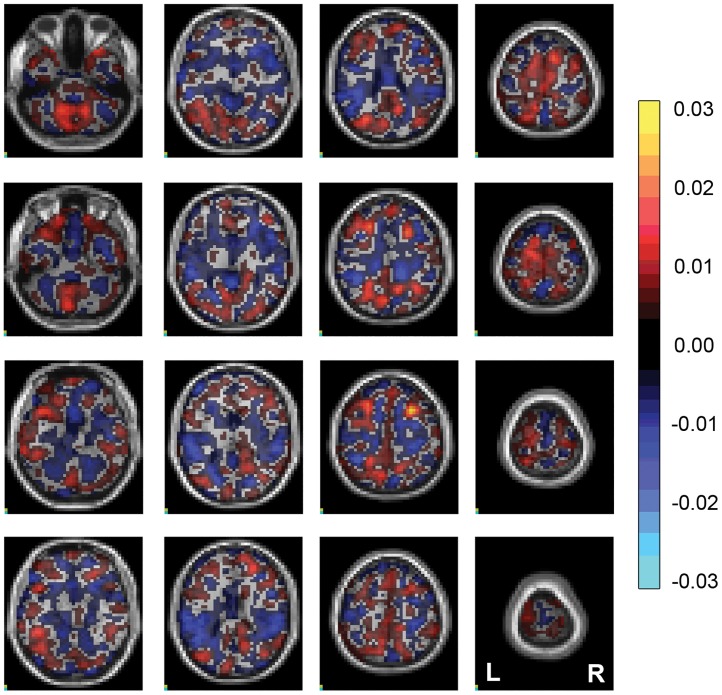
Weight maps for the prediction of the principal component 1. The slices contain the weight maps overlaid onto a standard MRI volume. Areas in red represent positive weights (metabolism correlates directly with the component) and areas in blue represent negative weights (metabolism correlates inversely with the component). L = left, R = right.

The weight map generated for the prediction of PC1 can be seen in Fig 4. As expected with a multivariate approach, there is a diffuse pattern of positive and negative weights related to the GPR prediction.

## Discussion

In this study, we aimed to investigate the relationship between the metabolic activity of the brain, as measured by 18F-FDG PET/CT imaging during resting state, and cognitive abilities in NF1 patients. We started by comparing the NF1 group’s cognitive performance with that of a control group and found that the NF1 group’s cognitive performance tended to be poorer, with medium to high differences compared to the control group, despite low statistical power to detect low and medium differences. Intelligence and verbal short-term memory were the most impaired functions, reflecting an overall mild intellectual inefficiency and a more heightened difficulty with maintenance of information than with active process of manipulation in working memory in this NF1 sample. Only small differences were observed between groups in long-term episodic memory, verbal fluency, processing speed, interference control, and planning. These results may be due to the variable performance in specific cognitive measures among the NF1 subjects, which might reduce disparities between groups in our study. Nevertheless, as expected, the NF1 sample had an overall poorer cognitive performance compared to controls.

The next step was to analyze whether the pattern of metabolism could predict the cognitive performance of patients. We started by processing images in order to reduce the effect of age on the metabolism measured at each voxel. This step is important because the impact of age must be taken into account when applying a voxel-based analysis on FDG-PET brain images. Chugami et al described a rapid increase in brain metabolism in early childhood, reaching a peak at 4–9 years of age, with a slow decrease to adult levels at the later part of the second decade [47]. A more recent study of children between 11 months and 16 years showed increasing uptake occurring through to age 16 years [48]. In adulthood, global cerebral metabolic rates of glucose slowly decline with age, with a reduction of about 12–13% between ages 20 and 70 years [49]. Although the relationship between brain metabolism and age in our sample ranging from 8 to 44 years is likely not a linear one, in the absence of a consensual model for controlling age effects, we believe the linear approach is still preferable over no control for age.

PCA was performed on the 17 neuropsychological tests because many of tests overlap in the cognitive processes they measure. The principal components are a form of “summarizing” the neuropsychological data. By performing GPR only on the components that explain most of the variance, one can reduce the amount of tests performed and therefore increase statistical significance. Since the first three components explain 65,3% of the variance in the neuropsychological data, GPR was used to individually predict each one of these components. To ensure that the predictions made by GPR during the LOOCV were in fact due to an underlying metabolic pattern associated with the examined variable and not a random combination of features, the LOOCV was repeated a thousand times with permutation of the input variables. If the algorithm found a true relationship between brain metabolism and the examined variable to make the predictions, one would expect a less accurate prediction and therefore a lower correlation with the permuted values.

Only PC1 reached statistical significance after permutation testing, with a correlation of 0.926 (p<0.001). This component explained 34.8% of neuropsychological data variance and represents multiple cognitive functions. The main measures associated with this component were IQ (WAIS/WISC verbal and performance measures), verbal fluency and verbal memory, and it was strongly associated with a heterogeneous brain metabolic pattern. The weight map shows a diffuse pattern of positive and negative weights related to the GPR prediction. This might reflect the high heterogeneity of cognitive functioning seen in NF1 patients (2, 4, 5).

One must be careful when interpreting a multivariate feature map since all features contribute to the prediction. Furthermore, although positive weights reflect a direct relationship between brain metabolism and the predicted variable and likewise negative weights reflect an inverse relationship, the value of the weight does not represent the intensity of metabolism in a certain brain region. Finally, because the weight map represents the mean values across all LOOCV iterations, higher values do not necessarily correspond to a consistent high weight on each prediction.

Different MRI studies have focused on neuroanatomical correlates to the NF1 cognitive phenotype, including grey and white matter volume changes [50], white matter microstructure [8] and functional connectivity changes [51]. To the extent of our knowledge, no study so far has investigated the relationship between neuropsychological performance and resting state cerebral metabolism in NF1 individuals. In this regard, our results complement the scarce literature on functional brain imaging in NF1. We are aware of the study’s limitations such as the sample size, which is aggravated by the huge heterogeneity of the NF1 phenotype and therefore might restrict the generalization of our findings. Nevertheless, we avoided overfitting using LOOCV and confirmed the validity of the predictions using permutation testing. Further studies are necessary to confirm the results in a different and bigger group of patients.

In this study, we showed that it is possible to accurately predict measures of neuropsychological performance based on brain metabolism using a machine learning Gaussian Processes based algorithm in NF1 patients. This result suggests an underlying metabolic pattern that relates to more global/verbal aspects of cognitive functioning in this group.
